# *Mycobacterium neoaurum* Bacteremia in an Immunocompetent Patient in MICU: The First Reported Case in Qatar

**DOI:** 10.1155/crdi/3710417

**Published:** 2025-05-06

**Authors:** Alanoud Alshmarri, Noor Jasim, Hisham Elhiday, Bara Alqudah

**Affiliations:** Department of Internal Medicine, Hamad Medical Corporation, Doha, Qatar

**Keywords:** bacteremia, case report, immunocompetence, intensive care units, nontuberculous mycobacteria

## Abstract

**Background:** *Mycobacterium neoaurum* is a rare, rapidly growing nontuberculous mycobacterium that typically affects immunocompromised patients and is often associated with catheter-related infections.

**Case Presentation:** We describe a case of *Mycobacterium neoaurum* bacteremia in an immunocompetent male admitted to the MICU with severe intracerebral hemorrhage. Notably, the infection occurred in the absence of central venous access. The organism was identified using MALDI-TOF MS, and the patient responded microbiologically to a combination antibiotic regimen, although his neurological prognosis remained poor.

**Conclusion:** This is the first documented case of *Mycobacterium neoaurum* bacteremia in Qatar and one of the few reported cases globally in an immunocompetent patient without conventional risk factors.

## 1. Introduction

*Mycobacterium neoaurum* is a pigmented, rapidly growing NTM from the *Mycobacterium parafortuitum* complex. Infections are rare and usually occur in immunocompromised individuals, particularly in the setting of indwelling catheters. Here, we report a unique case of bacteremia in an immunocompetent patient without central venous access.

## 2. Case Presentation

A 53-year-old Indo-Aryan male, immunocompetent with well-controlled Type II diabetes mellitus and hypertension, was admitted to the Medical Intensive Care Unit with intracerebral hemorrhage extending into the ventricles and causing a midline shift. The patient underwent intubation and sedation, and an arterial line was inserted. In addition, the neurosurgery team placed an external ventricular drain.

Two weeks after admission, the patient developed a recurrent fever. The initial examination yielded unremarkable findings; consequently, empirical treatment with piperacillin/tazobactam was initiated. A comprehensive septic workup was conducted, including blood cultures from both the arterial and peripheral venous lines, as well as cerebrospinal fluid analysis. The results indicated elevated inflammatory markers, while CSF analysis was within normal parameters, and the cultures remained negative. Tests for tuberculosis in the sputum and CSF, as well as HIV screening, yielded negative results. Chest radiography revealed no infiltrates, and transthoracic echocardiography demonstrated no vegetation or intracardiac masses.

Despite treatment with piperacillin/tazobactam, the patient continued to experience pyrexia. On Day 7, peripheral blood cultures from the arterial and venous lines demonstrated *M. neoaurum* growth, which was identified using matrix-assisted laser desorption/ionization-time of flight mass spectrometry (MALDI-TOF MS). The infectious disease team was consulted, and piperacillin/tazobactam was discontinued. Empirical therapy with meropenem, amikacin, and moxifloxacin was initiated, peripheral lines were replaced, and culture samples were sent abroad for sensitivity testing.

Following the implementation of the new treatment regimen, the patient's fever resolved, inflammatory marker levels normalized, and blood cultures from the peripheral lines were negative within 1 week.

Despite microbiological improvement, the patient's neurological condition remained poor. He was tracheostomized and off sedation, with a Glasgow Coma Scale score of 3T/15 and bilaterally nonreactive pupils (3 mm). After 5 weeks of admission, given the lack of neurological recovery and poor prognosis, a Do-Not-Attempt-Resuscitation order was established in accordance with the family's wishes and medical consensus. The patient subsequently passed away later that day. The cause of death was deemed to be a consequence of his severe neurological injury.

The susceptibility test results were finalized approximately one and a half months after samples were sent (Table 1); however, by that time, the patient had already deceased. Consequently, the treatment was not modified based on the susceptibility profile. He received a total of 3 weeks of antimicrobial therapy prior to his passing.

## 3. Discussion

*Mycobacterium neoaurum* is a rapidly growing, pigmented, nontuberculous mycobacterium that belongs to the *Mycobacterium parafortuitum* complex. These organisms are widely distributed in the environment and commonly found in water, soil, and dust. Although human exposure to these bacteria is frequent, it rarely results in clinical disease, suggesting relatively low virulence in the general population [1]. The rarity of clinical infections, despite environmental ubiquity, adds a diagnostic challenge and underlines the need for clinician awareness of atypical presentations.

*Mycobacterium neoaurum* infections are primarily reported in immunocompromised individuals, particularly those with multiple comorbidities, malignancies, chronic lung disease, long-term or recurrent antibiotic exposure, indwelling catheters, prosthetic valves, or other foreign devices. Although these infections are uncommon, they may also occur in immunocompetent individuals, indicating that under specific conditions—such as prolonged hospitalization or critical illness—the host immune response may still be overcome. This pathogen can manifest in various clinical forms, including sepsis, pulmonary involvement, and skin infection. Literature reviews suggest that catheter-related bloodstream infections are the most frequent presentation, whereas meningoencephalitis, endocarditis, and other systemic manifestations remain rare [2].

Laboratory identification of *Mycobacterium neoaurum* can be achieved through routine aerobic blood cultures on Lowenstein–Jensen agar, which typically yields growth within 5 days at temperatures ranging from 25°C to 35°C. However, these conventional methods may lack specificity. Advanced microbiological techniques, such as high-performance liquid chromatography (HPLC), sequencing of the 16S rRNA gene, and MALDI-TOF MS, offer rapid and accurate species identification. In this case, MALDI-TOF MS was used for definitive identification [3]. The application of such tools is a strength of this case, contributing to prompt microbiological diagnosis and appropriate antimicrobial decision-making.

Although there are no standardized guidelines for treating *Mycobacterium neoaurum* infections, a combination of antimicrobial agents, including macrolides, fluoroquinolones, and aminoglycosides, is generally advocated based on prior case reports and susceptibility profiles. Device removal, when applicable, is essential for preventing relapse and reducing the risk of resistance [4, 5]. In our case, although the patient did not have a central venous catheter, all peripheral lines were replaced as a precautionary measure. Empirical therapy with meropenem, amikacin, and moxifloxacin was initiated in accordance with literature-based recommendations. The patient showed a microbiological response, with resolution of the fever and normalization of inflammatory markers. Blood cultures turned negative within 1 week of initiating therapy, supporting the scientific rationale for the antibiotic regimen despite the absence of immediate susceptibility data.

*Mycobacterium neoaurum* was first isolated from soil and described in 1972. The first case of human infection was reported in 1987, involving a patient with metastatic ovarian cystadenocarcinoma who received total parenteral nutrition via a Hickman catheter [6]. Since then, the organism has been increasingly recognized as a rare opportunistic pathogen, particularly in device-related infections. The global literature remains scarce, especially in immunocompetent individuals. To date, only five cases have been described in such hosts, including one with bacteremia, one with pulmonary involvement, and three with cutaneous infections [7–11]. The first case of *Mycobacterium neoaurum* bacteremia in an immunocompetent patient was reported in 2016 in the United States, involving a woman with diabetes mellitus and a peripherally inserted central catheter. Despite treatment with ciprofloxacin and doxycycline, the patient was lost to follow-up, and the outcomes remained undocumented [7].

In our case, the patient had well-controlled Type II diabetes mellitus and hypertension but no known immunosuppressive condition or central venous access. He developed *Mycobacterium neoaurum* bacteremia while being managed in the ICU for severe intracerebral hemorrhage. The infection responded well to empiric antibiotic therapy with prompt clearance of bacteremia; however, the patient's neurological condition remained unchanged, and he ultimately passed away. Susceptibility testing, finalized approximately 6 weeks after the initial cultures, was not available during his treatment course and thus did not inform the regimen used.

This case is scientifically significant for several reasons. First, it underscores the ability of *Mycobacterium neoaurum* to cause bloodstream infections in immunocompetent individuals, even in the absence of traditional risk factors. Second, it demonstrates the utility of advanced microbiological diagnostics, such as MALDI-TOF MS, for identifying rare NTMs. Third, it highlights both the strengths and limitations of empirical therapy in the absence of susceptibility data. To the best of our knowledge, this is the first case of *Mycobacterium neoaurum* bacteremia reported in Qatar, contributing to the global understanding of its clinical spectrum.

## 4. Conclusions

Infections caused by *Mycobacterium neoaurum* are uncommon and often associated with immunocompromised states or indwelling medical devices. However, this case illustrates that severe infections can occur in immunocompetent individuals without these risk factors. Clinicians should maintain a high index of suspicion for rare NTMs in critically ill patients, particularly those with persistent bacteremia. Early identification using advanced diagnostic tools and timely empirical combination therapy can result in favorable microbiological outcomes. Nevertheless, individualized treatment decisions must consider the patient's overall clinical context and the delayed availability of susceptibility results.

## 5. Patient Perspective

Due to the patient's poor neurological status following severe intracerebral hemorrhage, he remained unconscious throughout the course of hospitalization and treatment. As such, he was unable to share his perspective or experience regarding the diagnosis or therapy.

The family was updated regularly about his condition and informed about the identification of a rare bloodstream infection and the rationale behind the antimicrobial treatment plan. They expressed appreciation for the continued efforts made to manage both the infection and his critical neurological condition, despite the overall poor prognosis.

## Figures and Tables

**Table 1:** Antibiotic susceptibility results for *Mycobacterium neoaurum*.

Organism: *Mycobacterium neoaurum*		
Antibiotic	MIC (mcg/mL)	Interpretation
Cefoxitin	8	S
Imipenem	0.25	S
Clofazimine	0.06	
Ciprofloxacin	≤ 0.12	S
Moxifloxacin	0.03	S
Clarithromycin	8	R
Amikacin	≤ 1	S
Tobramycin	2	S
Doxycycline	≤ 0.12	S
Tigecycline	≤ 0.03	
TMP/SMX	≤ 0.25/4.75	S
Linezolid	≤ 1	S

*Note:* The table summarizes the antibiotic susceptibility of *Mycobacterium neoaurum*, listing the minimum inhibitory concentration (MIC) values in micrograms per milliliter (mcg/mL). Each antibiotic is interpreted as either susceptible (S) or resistant (R) based on these values. One of the antibiotics listed is trimethoprim/sulfamethoxazole (TMP/SMX), which is presented with its combined MIC values.

## Data Availability

Data sharing is not applicable to this article as no datasets were generated or analyzed during the current study.
